# Toward Improving Human Training by Combining Wearable Full-Body IoT Sensors and Machine Learning

**DOI:** 10.3390/s24227351

**Published:** 2024-11-18

**Authors:** Nazia Akter, Andreea Molnar, Dimitrios Georgakopoulos

**Affiliations:** School of Science, Computing and Engineering Technologies, Swinburne University of Technology, Melbourne 3122, Australia; naziaakter@swin.edu.au (N.A.); dgeorgakopoulos@swin.edu.au (D.G.)

**Keywords:** wearable sensors, internet of things, machine learning, work activity recognition, worker training

## Abstract

This paper proposes DigitalUpSkilling, a novel IoT- and AI-based framework for improving and personalising the training of workers who are involved in physical-labour-intensive jobs. DigitalUpSkilling uses wearable IoT sensors to observe how individuals perform work activities. Such sensor observations are continuously processed to synthesise an avatar-like kinematic model for each worker who is being trained, referred to as the worker’s digital twins. The framework incorporates novel work activity recognition using generative adversarial network (GAN) and machine learning (ML) models for recognising the types and sequences of work activities by analysing an individual’s kinematic model. Finally, the development of skill proficiency ML is proposed to evaluate each trainee’s proficiency in work activities and the overall task. To illustrate DigitalUpSkilling from wearable IoT-sensor-driven kinematic models to GAN-ML models for work activity recognition and skill proficiency assessment, the paper presents a comprehensive study on how specific meat processing activities in a real-world work environment can be recognised and assessed. In the study, DigitalUpSkilling achieved 99% accuracy in recognising specific work activities performed by meat workers. The study also presents an evaluation of the proficiency of workers by comparing kinematic data from trainees performing work activities. The proposed DigitalUpSkilling framework lays the foundation for next-generation digital personalised training.

## 1. Introduction

A work skill is the ability of a worker to perform a specific task that usually requires different modes of action and body movements with frequent repetition [1,2]. Training is vital for any worker to enhance his/her proficiency in skills that are required to proficiently complete the assigned task [3]. Therefore, work training helps individuals acquire and refine their skills, leading to improved proficiency and productivity [4]. A crucial step in training a worker is to identify the individual’s current proficiency in performing any specific work activities required for the task he/she is assigned to complete [5]. In traditional training, a human trainer is primarily responsible for recognising and assessing the work activities of the trainees [6]. However, the trainer’s abilities to observe and analyse information from the trainees are limited, and the trainer’s evaluations of the activities and skills of each trainee are often subjective. These challenges are due to well-understood human limitations in observing and analysing vast amounts of information from trainees, which leads to poor skill proficiency improvement [7]. Moreover, traditional training often uses a one-size-fits-all approach [8]. Whereas, in addition to each trainee’s baseline proficiency level, individual trainees also exhibit differences in their capacity to acquire new information, as well as in their preferred learning styles and cognitive approaches to skill acquisition [9]. By not considering each trainee’s skill proficiency level in performing specific activities, they undergo unnecessary training or are not trained enough to perform the activities that are required to improve their skills [10]. In current training practices, trainers typically rely on easily trackable parameters, such as distance covered, materials produced, or time taken to complete tasks [3]. Several studies have used production output, faster completion time and quality of work as metrics for measuring work skill proficiency [11]. However, in improving work proficiency, these measurements are not always sufficient. The movement patterns of workers, particularly hand and other body parts movements, can significantly contribute to proficiency [12]. To address these issues, this paper proposes DigitalUpSkilling, a novel wearable IoT-ML-based framework for digital and personalised worker training. DigitalUpSkilling supplements the human trainer’s partial observations and related subjective skill evaluation of the trainees, with comprehensive observations performed by IoT sensors [13] and smart ML-based models for recognising and evaluating skill proficiency, providing a data-driven and personalised worker training solution.

For any sensor-based training, the first and most crucial step is human activity recognition. Related work on human activity recognition (HAR) can be classified as either sensor-based or vision-based HAR. Existing vision-based HAR solutions typically analyse camera images [14] and thermal images [15], or they propose human pose recognition algorithms [16]. As such, research typically focuses on detecting any activity, and the accuracy of vision-based HAR algorithms is usually limited in real-world work environments [17]. Sensor-based HAR is gaining momentum due to recent advancements in wearable sensor technology and accuracy [18,19,20,21]. Currently, wearable sensors can collect data from real-life environments without any visual obstruction and with great accuracy. Furthermore, no related study has used workers’ kinematic data from body movements during work activities to improve worker skills and work performance. Some related work has proposed combining sensors and ML for improving performance in sports [22,23,24]. Another advantage of using sensors is the easy creation of digital twins, which have proved to be useful in digitalising manufacturing activities [25], but currently, their use is very limited in real-world activity recognition. Finally, while some related works [26] on AI-based personalised worker training showed improved training and worker performance, they did not consider improving workers’ skills in physical work activities.

To illustrate the proposed DigitalUpSkilling framework and its benefits, the paper presents a study on training meat processing workers in a real-life production environment. The meat processing industry is one of the biggest export industries in Australia [27], and a significant portion—57%—of the production cost is attributed to workers’ expenses [28]. Inefficiency in training can lead to higher labour costs and less productivity. By enhancing training methods, the proposed framework can reduce these inefficiencies, enabling workers to perform their tasks faster and with greater accuracy, ultimately reducing labour costs and improving overall productivity. One key aspect of the DigitalUpSkilling framework is its evaluation of work activity recognition accuracy in meat processing tasks; then, it compares the performance attributes of the recognised activities. However, challenges such as limited datasets and a small number of participants complicate its study. Additionally, like other real-world job activities, these tasks are not time-bound, increasing the likelihood of activity class imbalance. To overcome these limitations, the study proposes a hybrid GAN-ML activity classification model that leverages generative techniques to augment data and enhance classification performance.

In summary, the novel contributions of this paper are the following:

- Digital twins of workers that continuously synthesise avatar-like kinematic models of the activities for each worker being trained. In doing so, it uses wearable sensors that observe how individual workers perform physical work activities;
- A hybrid GAN-ML work activity recognition model for recognising the types of work activities each worker performs;
- Skill proficiency recognition analysis for evaluating how well each trainee performs specific work activities and the overall task he/she is responsible for;
- An industry study that illustrates how highly accurate work activity recognition GAN-ML models can detect complex meat processing activities using body movement data from full-body wearable IoT sensors.

The remainder of the paper is organised as follows: Section 2 presents related work on IoT-GAN-ML-based HAR and skill assessment. Section 3 describes the proposed DigitalUpSkilling framework. Section 4 presents a study on activity recognition involving complex work activities performed by meat processing workers. Section 5 discusses the performance of the activity recognition GAN-ML model in the study, while Section 6 discusses work skill proficiency measurement from sensor data and digital twins. Section 7 concludes the paper and provides directions for future research.

## 2. Related Work

As discussed in Section 1, the DigitalUpSkilling framework involves steps for automatically recognising and measuring the proficiency of individual workers conducting specific work. The steps include collecting body movement data, synthesising kinematic digital twins of the workers being trained, and training and using GAN-ML models for work activity classification and for skill assessments. This section provides an overview of the relevant literature related to these steps.

IoT sensors for digitally capturing human movement from work activities: Because of its versatile nature and performance, wearable sensor-based technology is now used in various fields, including smart homes, healthcare, and sports [29,30,31,32]. Wearable IoT sensors gained popularity in work activity recognition because they can easily be integrated into garments and accessories or even directly attached to the body, enabling unobtrusive monitoring during activities [33]. Also, IoT sensors are great tools for human activity recognition for users who are involved in rigorous activity and movement [34,35]. The accelerometer and gyroscope in an IoT sensor can connect body movement data from individuals. These sensors can capture kinematic data from work activity and create a kinematic model. The abundance of data collected by sensors enables the tailoring of training programmes according to individuals’ specific needs and capabilities, thereby optimising their proficiency during training activities [23,36]. Recently, smartphones and wristbands have become popular tools for activity recognition in real-world environments due to their portability and the ability to collect continuous sensor data [37,38]. These devices, equipped with accelerometers, gyroscopes, and heart rate sensors can effectively monitor physical activities, such as walking and running, as well as even stress, providing valuable data for health and fitness applications when combined with ML models [39]. However, there are limited studies focusing on body movement metrics such as joint angles, smoothness of movements, and abduction, which are crucial to work proficiency.

Moreover, several studies have used IoT sensors on participants’ hands to analyse their movement [35,40], and other studies [32,34,35,41] have mounted sensors on other body parts of participants, such as on the sacrum and shoulder. Studies show that placing a single sensor on the human body allows for the detection of movement with that particular body part [42]. In contrast, full-body sensors allow for the capture of full-body movements and, hence, can provide more precise feedback about body movement and proficiency with a full-body kinematic model [42,43,44], which is important for work activities.

Research has also shown good accuracy in terms of recognising human activity from sensor data [45,46,47]. For posture recognition of construction workers, Subedi et al. [48] used a depth sensor camera to collect movement data and an ML algorithm for work activity classification.

IoT-sensor-based digital twins for capturing human movement: The integration of the IoT and digital twins for human training has created new possibilities for data-driven and personalised learning. By gathering real-time data from IoT devices, including wearable sensors, digital twins—a virtual representation of a physical entity—are produced, enabling continuous monitoring and feedback on performance [49]. Though the use of digital twins is not new in the field of manufacturing in the Industry 4.0 era [50,51], in human training, particularly in physically intensive activities, it is underutilised. Digital twins have the ability to track detailed human body movements in real time [43], provide insights into skill proficiency [52], and contribute to next-generation training [43]. IoT devices, like inertial measurement units (IMUs) or smart wearables, stream real-time data to digital twins, representing and analysing human movements in real time [42], thus facilitating adaptive training environments.

GAN-ML-based work activity classification: From IoT sensor data, activities can be recognised and measured using machine learning algorithms [21]. ML-based HAR can detect physical activity, identify areas for improvement, and suggest actions for improvement [53]. The last decade witnessed ML-based activity recognition in different domains, including smart homes, healthcare, rehabilitation, security, and the sports industry, which includes both simple and complex physical activities by humans [18,19,20,21].

In a literature review [54] of daily activities, such as running, jogging, eating, and biking, various ML-based HAR models achieved high accuracies of 80-98%. In another literature review, Yadav et al. [55] present a summary of seven studies that used wearable IoT sensors with different machine learning algorithms, such as random forest (RF) and support vector machine (SVM), showing accuracies of 72% to 98%. Research by Forkan et al. [56] on the meat industry used IoT- and ML-based models to recognise work activities, resulting in good accuracy in activity recognition. Subedi et al. [48] used an ML algorithm for activity classification on construction workers but in a non-work environment. In addition, other studies exist on ML-based activity recognition for workers [57,58]. So, from these studies it can be concluded that human activity recognition models, such as RF and SVM, are widely used and provide good accuracy.

To address the issue of small datasets for human activity recognition, some studies used the generative adversarial network (GAN), conditional generative adversarial networks (CGAN), etc., as data augmentation models [59,60]. In addition to GANs, ML models, such as the synthetic minority oversampling model (SMOTE) [61,62] and adaptive synthetic sampling approach (ADASYN) [63], have been applied to mitigate the challenges of imbalanced datasets of real-world scenarios. Among these approaches, the combination of GAN and SMOTE often outperformed other models [64]. Furthermore, to enhance the data quality and remove overlapping instances, techniques such as Tomek links and edited nearest neighbours (ENN) are frequently used [65,66].

ML-based skill proficiency measurement and assessment: Several studies have been conducted to understand human proficiency using IoT and ML models, mainly in sports. In one study, Su and Chen [22] used IoT and AI models to predict the scores of basketball players and support the team’s decision-making process. The data used in the research were from an existing dataset of NBA league players, and proficiency was predicted based on the players’ salaries. Another study [67] used an ML model to predict and evaluate the proficiency of female handball players using their physiological characteristics, such as BMI and height. Pappalardo et al. [68] evaluated the proficiency of soccer players using ML models. A systematic review by Lam et al. regarding proficiency in technical skills in surgery by ML models showed good accuracy [69]. However, these studies focused on external factors like salaries and physiological characteristics rather than measuring their proficiency based on their actions or body movements when performing the tasks.

Machine learning (ML) models are increasingly being used to analyse complex biomechanical data, such as flexion, abduction, and acceleration of body parts, which are traditionally overlooked during training because of the limitations of human observation [70]. ML can process large datasets generated from sensors to extract meaningful patterns and insights about movement efficiency, enabling real-time feedback and performance improvement [71]. By leveraging ML, these methods can provide precise, data-driven insights that help optimise worker training and enhance proficiency [72].

AI and IoT solutions for personalised training frameworks for workers: Fraile et al. [73] proposed a methodological framework for personalised training programmes for industry workers using artificial intelligence (AI) and neural language processing (NLP). The study focused on the internal conversations of workers in the workplace and determined their skills from these conversation. One study [74] proposed an AI-based framework for personalised employee training to improve the overall learning models and abilities. In the study, a questionnaire and feedback were collected from employees. Then, the NLP model was used for skill extraction and assessment of the questionnaire and feedback. Butean et al. [75] proposed an AI- and VR-based solution for improving training methods for industry workers, where they proposed a learning platform for training humans, including industrial workers.

Research gaps: Related work that employs wearable IoT sensors for work activity recognition is in the early stages. Existing GAN-ML models for work activity recognition and skill proficiency measurement have been trained with work data collected in laboratories, not real-life production settings. Similarly, there are currently no studies using GAN-ML models that are trained and developed using human movement data from work-related activities specifically to assess work skill proficiency. Finally, to the best of our knowledge, we could not find any frameworks or studies that combined IoT, GAN, and ML models for work activity recognition and work skill proficiency measurement for the training of workers.

## 3. DigitalUpSkilling Framework for Digital Personalised Training

**Figure 1:**
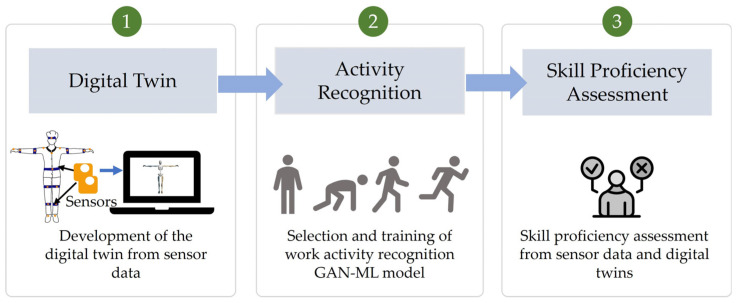
DigitalUpSkilling framework.

The proposed DigitalUpSkilling framework includes the following three major parts, as depicted in Figure 1: (1) development of digital twins (i.e., kinematic models) of each trainee from sensor data observations that are produced by the wearable sensors he/she is wearing; (2) selection and training for work activity recognition using a hybrid GAN-ML activity classification model; and (3) development of the skill proficiency assessment ML model. Section 3.1, Section 3.2 and Section 3.3 present these parts of the DigitalUpSkilling framework in further detail.

### 3.1. Data Collection Using Wearable IoT Sensors and Generation of Workers’ Digital Twins

As work activities have many variations without set patterns, it is difficult to distinguish them. Therefore, to capture work activities, the DigitalUpSkilling framework employs full-body wearable IoT sensors that collect kinematic data from inertial measurement units (IMUs) attached to different parts of each worker’s body. The sensor data observations include body joint rotations, movements, and related accelerations that determine a participant’s orientation, position, and movement in real time.

The DigitalUpSkilling framework uses all of the kinematic data that are collected by these sensors on each worker to generate real-time digital twins of their movements. The workers’ digital twins are used to capture and observe the movements that are necessary to perform specific work activities and the sequence of such activities in the completion of tasks. Ground truth is provided by a camera that records the training and corresponding work session, and the resulting video is then used for data labelling during ML training.

### 3.2. Work Activity Recognition

**Figure 2:**
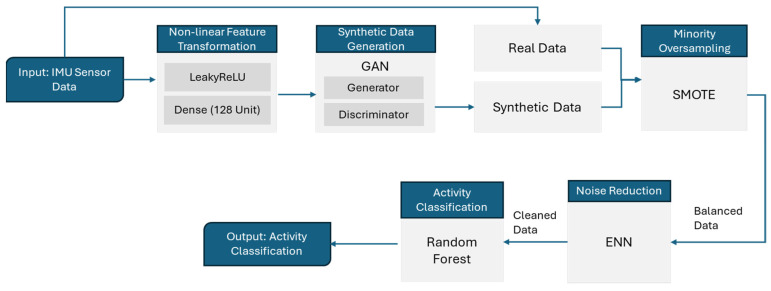
Hybrid GAN-ML activity classification.

The DigitalUpSkilling framework proposed a hybrid GAN-ML activity classification model that can be trained to recognise specific work activities from human body movement (Figure 2). First, the accuracies of the ML models are evaluated on the original sensor data. Then, the classification accuracies for combinations of sensors on different body parts, as well as the combination of sensors, are evaluated. Then, the proposed model’s GAN uses a generator and discriminator to generate synthetic data and distinguish synthetic data from original sensor data, respectively. This process enhances the size of the collected data set. After, to balance out any kind of class imbalance, the SMOTE model is used, which conducts minority oversampling and balances the dataset. In the next step, ENN is used to filter out noise to decrease misclassification in the nearest classes with the closest values. Finally, the RF model is used to classify the activity. In the proposed model, supervised ML models are trained to recognise specific work activities that are typically performed by workers who work on the same activities. The proposed classification model takes IMU sensor data as inputs and analyses the kinematic data on the body parts of the trainees while they are performing work activities and classifies their activities.

### 3.3. Skill Proficiency ML

**Figure 3:**
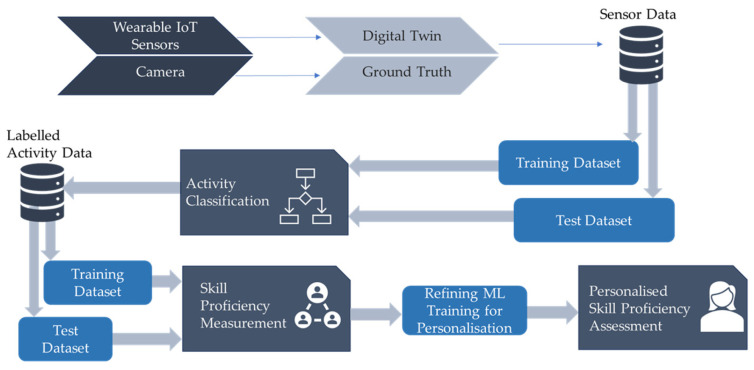
Skill proficiency assessment.

The DigitalUpSkilling framework uses ML-based work performance classification and assesses work skill proficiency related to physically intensive jobs. The training of the skill proficiency ML models is achieved as follows: (1) first, it uses the kinematic data collected from digital twins of all workers who perform that work activity; (2) using the proposed GAN-ML-based work activity recognition model, it divides this comprehensive dataset into work activity-specific datasets (each of which contains kinematic data from all workers performing that type of activity); (3) finally, for each type of activity detected by the work activity recognition ML model, it trains the model with data from workers that performed the best in this activity type (e.g., produced more of the product, fewer defects, and better quality products, or a combination of these) to classify proficiency. This approach to proficiency assessment using the DigitalUpSkilling framework is depicted in Figure 3.

For certain work tasks, the skill proficiency model may need to be tailored to each specific activity type; in other cases, a single model may suffice for assessing skill proficiency across various activities. We define the test data and training dataset for activity recognition using ML models. This approach can be refined further to account for the activity’s difficulty level or the gender, age, and past work experience of the workers being trained. This enables a more personalised and accurate skill proficiency assessment, allowing for the customisation of ML training models to better evaluate individual skill levels.

Furthermore, the ground truth is updated whenever a new dataset is added to this framework. Hence, the framework will help improve the overall training approach for industrial workers and make the training and assessment digitalised and personalised.

## 4. Study of Work Activity Recognition for Meat Processing Activities

We conducted a real-world study on the meat processing industry based on the proposed DigitalUpSkilling framework. This section depicts the design of the study.

**Figure 4:**
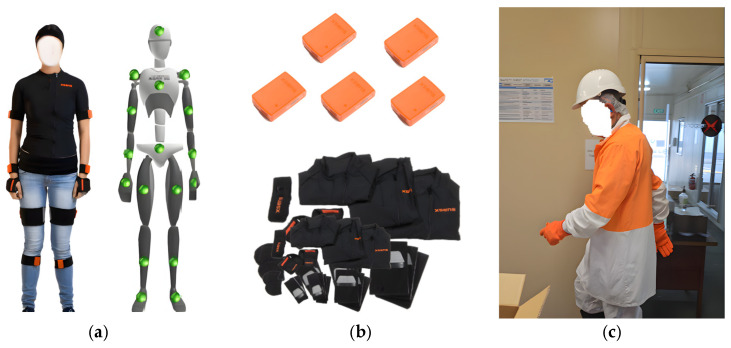
(**a**) Placement of sensors; (**b**) sensors and straps; (**c**) alignment of sensors with the participant’s movements.

Sensor selection for data collection: The study used the Movella Awinda Suit [76], which has 17 IMU sensors. It provides data in 60 Hz from a 100 m range. The 17 IMU sensors were placed on the participants’ wrists, forearms, upper arms, shoulders, feet, lower legs, and upper legs, as well as on hips, chests, and necks (Figure 4), which provided a full-body kinematic model and could capture the movement of any type of work activity.

**Figure 5:**
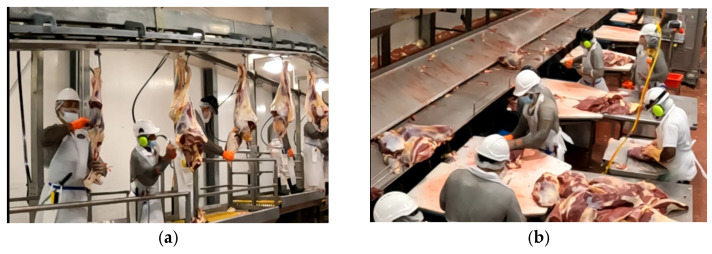
Work environment for the data collection: (**a**) boning area; (**b**) slicing area.

Participants and datasets: In this study, we had two male participants with more than fifteen years of work experience who are experienced trainers. We had fewer participants in this study, as obtaining such data from participants working in real work environments is challenging (Figure 5).

Moreover, no public datasets contained data on work activities in real work environments that were suitable for our study [77]. The entire data collection procedure was carried out inside the meat processing factory while participants performed their regular meat processing tasks.

The participants performed two repetitions with two different knives for each activity set, boning and slicing, to achieve a balanced dataset with different sets of body movements. Hence, the study had eight sets of activity data from the participants. We asked participants to use knives with varying levels of sharpness during the data collection to obtain broad variations in body movements and actions to ensure that the study included diversified data on real-work activities. The activities were based on two different meat processing plant assembly lines. Each activity set with different knives was performed for 5 to 15 min.

Activity selection: To incorporate real-life data, we chose meat processing workers involved in work activities such as boning and slicing meat in a meat processing plant. Both are crucial meat processing activities involving specific types of complex physical activities and techniques.

Boning: Boning is the removal of bones from large meat cuts. This physical activity requires steady hand control and attention to detail. Meat processors utilise various tools, such as knives and cleavers, to carefully separate meat from bones. They also trim excess fat and connective tissue. Boning requires repetitive motions to extract meat from the bones efficiently.

Slicing: Slicing involves cutting meat into thin, uniform slices. This task demands repetitive movement of the hand and body of meat workers. Different knives are used to create slices with a consistent thickness. Like boning, slicing also requires steady hand control and attention to detail.

**Figure 6:**
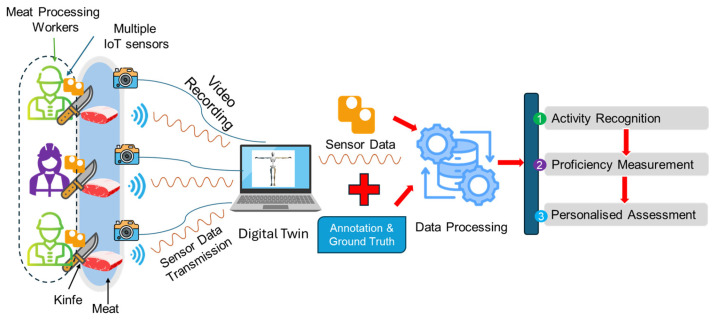
Dataflow of the study.

Data Collection: For collecting data, 17 IMU sensors were mounted on workers, using straps and a body vest. Then, they put on their uniform, performed the calibration, and entered the meat processing area to perform their work activities. We collected sensor data on the following activities they performed: boning and slicing. Sensor data were transmitted via Bluetooth to a computer system, where digital twins visualised worker movements in real time. Movements were also recorded using a video camera. The overall setup, data collection process, and data processing are presented in Figure 6.

**Figure 7:**
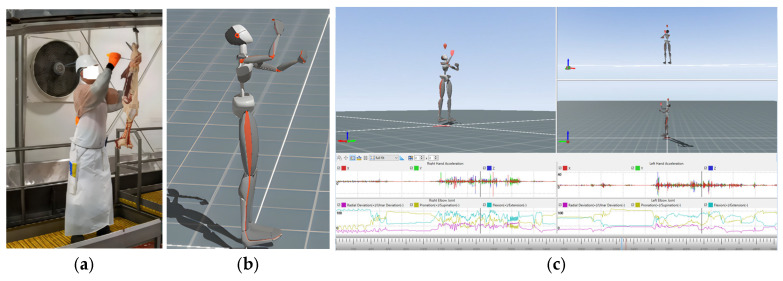
(**a**) Worker performing boning; (**b**) worker’s real-time digital twin; (**c**) digital twins showing body movements along with real-time graphs of the joint’s movements.

Digital twins from IMU data: Initially, we took the participants’ body measurements and placed the sensor as depicted in Figure 4. For initialisation of the digital twins representation, we calibrated the sensors before the worker started their regular work activity. We collected data from wearable full-body IoT sensors, which were recorded using the real-time digital twins created with MVN Analyse software (version: 2024.2.0) [76] (Figure 6). We also checked for magnetic interference, as this could possibly affect the quality of the digital twins and their live representations. The digital twins not only represented body movements via a 3D avatar, but we could also monitor any particular joint’s angle rotation, acceleration, and flexion, which are important factors in body movements and workers’ skills, as depicted in Figure 7c. The session was also recorded using a camera, which served as the ground truth for the digital twins. With the help of the calibration process, all sensors were properly aligned, and the digital twins’ representations were real representations of the participants’ body movements.

Data annotation from digital twins: We recorded each session using a video camera for record the ground truth. We annotated each microstep by synchronising the video with the real-time kinematic digital twins generated from the wearable full-body sensor data using MVN Analyse software. We synchronised the signals from each sensor, the video, and the associated timestamps to annotate data accurately.

Collected data included activities such as idleness, walking, steeling, reaching, cutting, slicing, pulling, placing/manipulating, and dropping. Each activity contained several single actions by different body parts, such as closing, reaching, opening, moving, unlocking, holding, cutting, spreading, releasing, dropping, picking, throwing, etc., as shown in Table 3. The dataset consists of 529,718 data samples from the IMU sensors, collected at a 60 Hz sampling rate.

The study found many low-level actions that make up complex work activities as shown in the Table 1.

### 4.1. Data Labelling

Activity labelling was conducted for each timestamp as given in the Table 2 and Table 3:

Data Preprocessing: After the annotation, we performed data preprocessing to clean raw sensor data and prepare them for activity classification. First, we removed missing data from the dataset using imputation techniques to fill in missing values, and we removed irrelevant or redundant features from the dataset, ensuring the data was clean and consistent. We applied data augmentation techniques using a generative adversarial network (GAN), with utilisation of a Leaky ReLU activation function and dense layers (128 units), to generate synthetic data and increase the size of the dataset, which initially contained 529,718 data samples from two participants. To address the issue of class imbalance, we then applied SMOTE (synthetic minority oversampling technique) to further balance the data by oversampling the minority classes, followed by edited nearest neighbours (ENN) to remove noisy or misclassified samples. The dataset was split into training and test sets with a 75/25 ratio using stratified sampling to preserve the class distribution. For each participant, a 969-dimensional matrix was created by the sensors, where 969 represents the features collected from 17 sensors for each data sample.

ML model selection for work activity recognition: For the study, we chose existing ML models that have been proven as effective at human activity classification. For this research, we chose RM, SVM, DT, and KNN for the work activity classification and then compared the accuracy of the ML models to find the most accurate one for this study. After computing the features, we split the training and test data to train the ML models. These ML models were trained on a labelled dataset comprising various work activities.

Feature selection: To classify data in the ML models, we extracted the 3D coordinates, velocity, acceleration, and rotational data for each of the 17 sensors. After, we selected the features of body parts that were active during the activities, and then the features that promised a high contribution to the recognition of the activity were selected. For each of the timestamps, we computed the mean (X‾) and standard deviation (sigma), where the cap X bar signifies the magnitude, and σ presents the variability. These values depict orientation-related data. The sensor readings for each variable were combined (X, Y, and Z) separately and the weighted sum of squares was calculated.

Then, the combined values for X, Y, and Z were calculated as follows:

(1) $$\mathrm{Sensor}\;\mathrm{Fusion}=\sqrt{\left(\sum_{i=1}^{n}X_{i}^{2}\right)+\left(\sum_{i=1}^{n}Y_{i}^{2}\right)+\left(\sum_{i=1}^{n}Z_{i}^{2}\right)}$$

Here

- *n* represents the total number of sensors;
- $X_{i}$, $Y_{i}$, and $Z_{i}$ represent the sensor readings for the ith sensor for X, Y, and Z, respectively.

This expression represents a form of vector magnitude or Euclidean norm that combines the contributions from all three components (X, Y, and Z) across the 17 sensors.

### 4.2. Data Preprocessing

Let the dataset be represented as $D={\left\{(x_{i},y_{i})\right\}}_{i=1}^{n}$, where $x_{i}$ is the feature vectors (sensor data), and $y_{i}$ is the corresponding activity labels.

- Handling Missing Values

For any feature vector, $x_{i}$, with missing data, the model applied an imputation function $Impute(x_{i})$, replacing the NaN values with the mean of the corresponding feature.

(2) $$x_{i}=Impute(x_{i})$$

- Train–Test Split

Split D into the training set, $D_{train}$, and the test set, $D_{test}$, at a 75−25% ratio.

### 4.3. Generating Synthetic Data Using GAN

- Generator: The generator G(z) takes a random noise vector, $z∼N\left(0,1\right),$ and outputs synthetic data, $x_{fake}$, in the same feature space as real data. The generator uses Leaky ReLU activations for hidden layers.
- Leaky ReLU activation

(3) $$f\left(x\right)=\left\{\begin{array}{c} x,\;\;\;\;\;\;\;x>0\\ \alpha x,\;\;\;\;\;x\leq 0 \end{array}\right.$$

where α is a small slope (e.g., 0.01) for negative inputs.

Generator output:

(4) $$x_{synthetic=G(z)}$$

- Discriminator: The discriminator, D(x), takes input, x, and outputs a probability, $D\left(x\right),$ that the input is real. It is trained to distinguish between real data, $x_{real}$, and synthetic data, $x_{synthetic}$.
- Discriminator loss for real data

(5) $$L_{real}=-log(D(x_{real}))$$

- Discriminator loss for fake data

(6) $$L_{synthetic}=-log(D(x_{synthetic}))$$

- Training GAN

The generator is trained to minimise the following:

(7) $$L_{G}=-log(D(G(z)))$$

The discriminator is trained to minimise the following:

(8) $$L_{D}=L_{real}+L_{synthetic}$$

- Synthetic data generation

After training, synthetic samples, $x_{synthetic}$, are produced by the generator, as follows: $x_{synthetic=G(z)}$.

### 4.4. Handling Class Imbalance with SMOTE

Let us assume $D_{minority}$ is a subset of $D_{train}$, representing the minority class data. Here, we use SMOTE to generate synthetic samples between the nearest neighbours of the minority class, as follows:

(9) $$x_{new}=x_{i}+\lambda \left(x_{i}-x_{i}\right)where\lambda ∼U(0,1)$$

This creates new synthetic data points, $x_{new},$ to balance the class distribution.

### 4.5. Data Cleaning with ENN

After SMOTE, we applied edited nearest neighbours (ENN) to remove noisy or misclassified data points.

For each sample,$\;x_{i}$, check its K-nearest neighbors, as follows:

(10) $$kNN\left(x_{i}\right)=\left\{x_{1},x_{2},\ldots x_{k}\right\}$$

If $x_{i}$’s label disagrees with the majority label in its neighbourhood, it removes $x_{i}$ from the dataset.

### 4.6. Classification with RF Model

Then, we trained the RF model, F(x), on the resampled and cleaned dataset, $D_{cleaned}$. RF consists of T decision trees, $F_{t}(x)$), each trained on a bootstrapped subset of the training data. Where the prediction for each sample, $x_{i}$, is given by the following:

(11) $$F\left(x_{i}\right)=majority_{vote}(F_{1}\left(x_{i}\right),F_{2}\left(x_{i}\right),\ldots ,F_{T}\left(x_{i}\right))$$

Here, each tree outputs a classification, and $F\left(x_{i}\right)$ is the final prediction.

Evaluation matrix of the study: We used accuracy, precision, recall, and the F1-score to evaluate various ML models’ performances. The following are the formulas for these metrics:

Accuracy: It presents the ratio of accurately classified samples to the total data samples in the model.

(12) $$\mathrm{Accuracy}=\frac{Number\;of\;Correct\;Predictions}{Total\;Number\;of\;Predictions}$$

Precision: It presents the ratio of correctly predicted positives to the total predicted positives, representing the accuracy of the positive predictions.

(13) $$\mathrm{Precision}=\frac{True\;Positives}{True\;Positives+False\;Positives}$$

Recall: It represents the ratio of accurately predicted positives to all data samples.

(14) $$\mathrm{Recall}=\frac{True\;Positives}{True\;Positives+False\;Negatives}$$

F1-Score: It presents the harmonic mean of the precision and recall of the data samples.

(15) $$F1-\mathrm{Score}=2\text{×}\frac{Precision\times Recall}{Precision+Recall}$$

In these formulas:

- True positives: Data correctly predicted as positive;
- False positives: Data incorrectly predicted as positive;
- False negatives: Data incorrectly predicted as negative;
- True negatives: Data correctly predicted as negative.

We also used the confusion matrix (CM) to evaluate the performance of the selected ML classification models. CM is a square matrix where the rows signify the true class labels, and the columns show the predicted activity labels. The diagonal element of the matrix depicts the percentage at which the predicted activity label matched the true activity label.

## 5. Performance of the Work Activity Recognition Model

**Figure 8:**
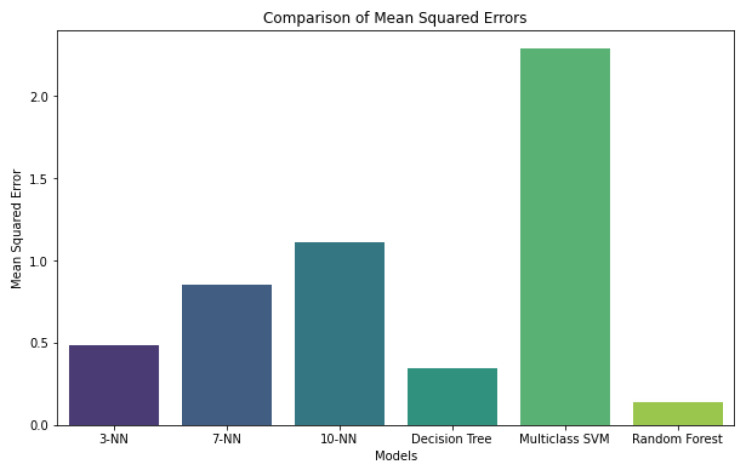
Comparison of the error rates of the different ML models.

We used several feature combinations and algorithms to obtain good accuracy in activity recognition. We used classification accuracy as the primary decision criterion and drew direct comparisons regarding classification accuracy. We compared the classification with all 969 features from 17 sensors for the full body movements. Our experiment shows that the RF model had the best classification accuracy for full-body sensor data. KNN and SVM showed higher error rates, as given in Figure 8, whereas the RF model had the least mean squared error (MSE). Therefore, we chose the RF model for the rest of the study to obtain the most accurate classification results.

Then, we compared the accuracy of the RF model using various single features and fused features for different combinations of sensors. The right hand is the most prominent part of the body that is actively involved in physical tasks. Later, we combined data from eight sensors that were placed on both hands and 17 sensors placed over the entire body. Table 4 represents the classification accuracy using RF, where we used single features, such as acceleration and magnitude, and then combined the features. The results show that the accuracy was slightly higher when using data from both hands and the full body rather than just the right hand. The most accurate results came from the pitch and roll data and the fused data on velocity, magnitude, and pitch and roll.

Table 5 represents the classification accuracy using RF, single, and combined features. The results show that the accuracy was different for different activity sets, such as boning and slicing, and for slicing, the accuracy was lower than for boning. When fusing different features, the accuracy increases, and the accuracy for boning and slicing is similar.

**Figure 9:**
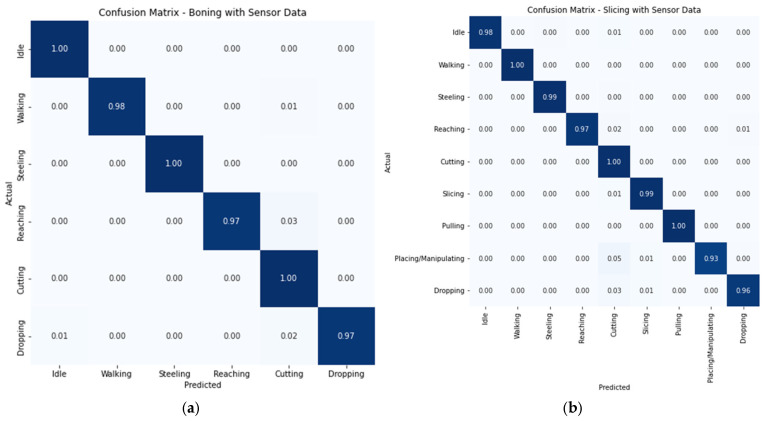
Confusion matrices: (**a**) boning; (**b**) slicing with pitch and roll from right-hand sensors.

Based on the results in Table 4 and Table 5, we used the random forest (RF) model for the confusion matrix of boning and slicing using pitch and roll data from four sensors on the right hand. The matrix depicts that the RF algorithm was effective for identifying simple activities, such as idleness, standing, and walking, as well as complex work activities, such as steeling, cutting, and slicing, which are recognised with high accuracy. This approach shows the accuracy of the ML model in recognising work activities for each participant using minimal features (Figure 9).

**Figure 10:**
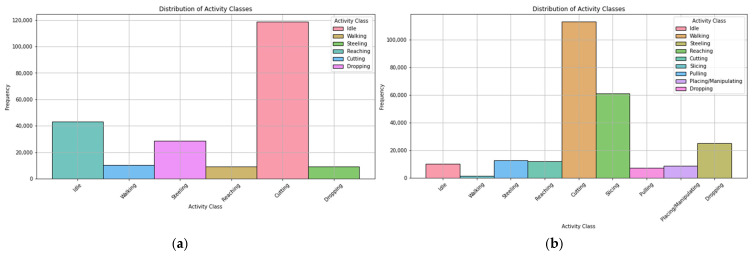
Distribution of the activity classification: (**a**) boning; (**b**) slicing.

After classification, the distribution of activity classes is given in Figure 10. The figure depicts that in boning, the number of data samples for cutting was significantly higher than for other activities. Similarly, for slicing, the data samples for cutting and slicing were much higher in number than the rest of the activities. Both figures indicate that there is an imbalance in the classes.

**Figure 11:**
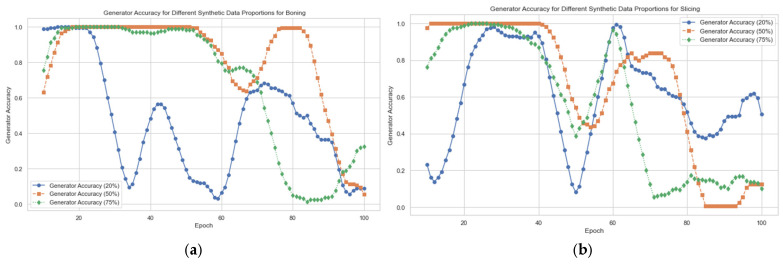
Accuracy of the GAN for different percentages of synthetic data: (**a**) boning; (**b**) slicing.

A total of 529,718 data samples were collected while performing boning and slicing. As the collected datasets show an imbalance in the classes as depicted in Figure 10, to make these data more robust, as well as to solve the data imbalance, we used a generative adversarial network (GAN) to generate synthetic data in different percentages: 25%, 50%, and 75%. The result show that 50% of the synthetic data yielded great accuracy with 60–80 epochs, as presented in Figure 11.

**Figure 12:**
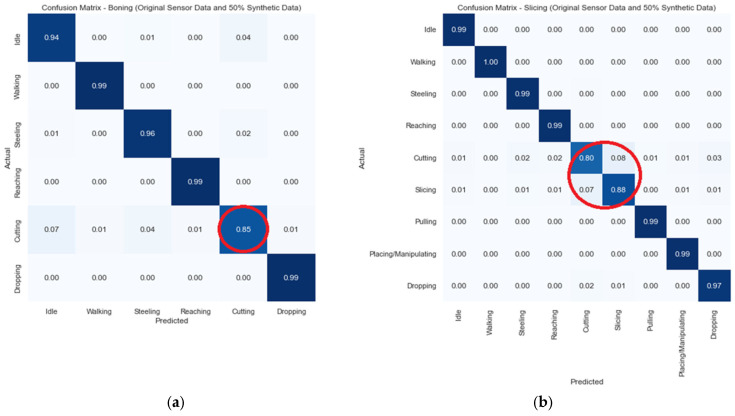
Accuracy of the GAN with different percentages of synthetic data (circled area showing drop in the accuracy): (**a**) boning; (**b**) slicing.

The model created synthetic data using a general GAN with a generator, and then we utilised a discriminator to distinguish between synthetic and real data. Along with the synthetic data generated by the GAN, to solve the class imbalance, the SMOTE was applied. The classification results are depicted in Figure 12.

**Figure 13:**
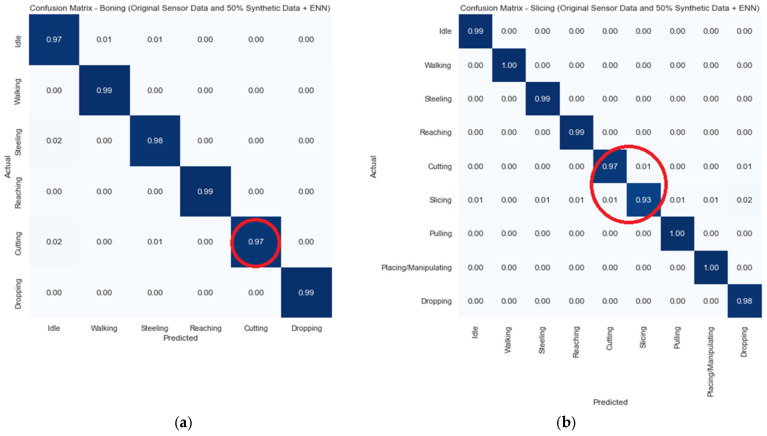
Classification accuracy with the GAN, SMOTE, and ENN (circled area showing improvement in the accuracy): (**a**) boning; (**b**) slicing.

The results in Figure 12 depict that after combing the synthetic data and combining the SMOTE, the accuracy of the major classes in both boning and slicing decreased. Especially for slicing activities, with 8% of the cutting class data being identified as slicing. Similarly, 7% of the slicing class data were identified as cutting. To overcome this misclassification, we applied another ML model, ENN, which removed nearest neighbours to decrease misclassifications. Hereafter, the classification results improved with an accuracy over 90%, as shown in Figure 13.

**Figure 14:**
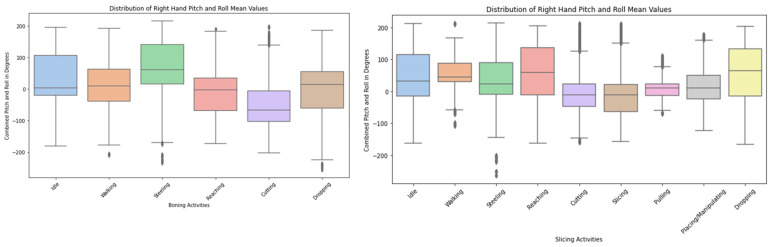
Distribution of right-hand pitch and roll mean (in degree).

After performing the activity recognition, we gathered preliminary findings of work proficiency analysis as per the proposed DigitalUpSkilling framework depicted in this paper. The preliminary analysis of body movement data demonstrates an influential use of the IoT-GAN-ML model to support the proposed framework. For example, Figure 14 shows that steeling, reaching, and dropping activities involve the most varied hand movements, with a wider range of pitch and roll values. In contrast, cutting, slicing, and pulling have more stable hand orientations, with pitch and roll values concentrated around the median.

The initial results from the study show that workers’ engagement, as well as the effectiveness of the tools used for work activities, can be determined from the proposed GAN-ML-based HAR. Figure 14 suggests a correlation between the sharpness and idle time of the workers, as using sharp knives allows participants to work less while maintaining their productivity. For instance, worker 2 with a sharp knife was more idle but remained more productive compared to when using a dull knife.

**Figure 15:**
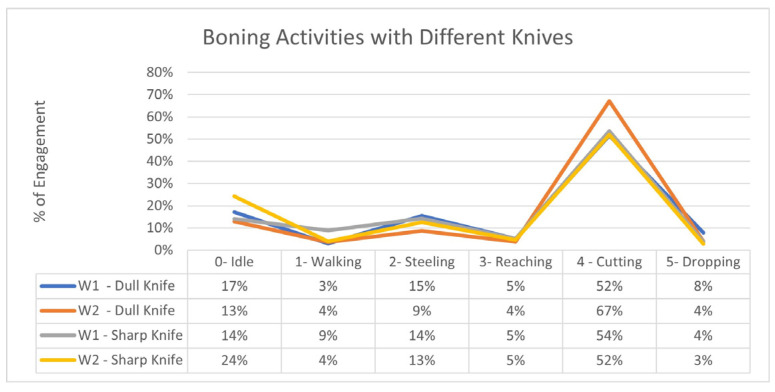
Comparison of the engagement in boning (W1: Worker 1; W2: Worker 2).

Furthermore, the model can also determine the effectiveness of the sharp tools. Figure 15 demonstrates that as the knives became dull, workers were exerting more effort and remained more engaged in cutting rather than slicing, as slicing mostly depends on the sharpness of the knife and requires less effort than cutting.

**Figure 16:**
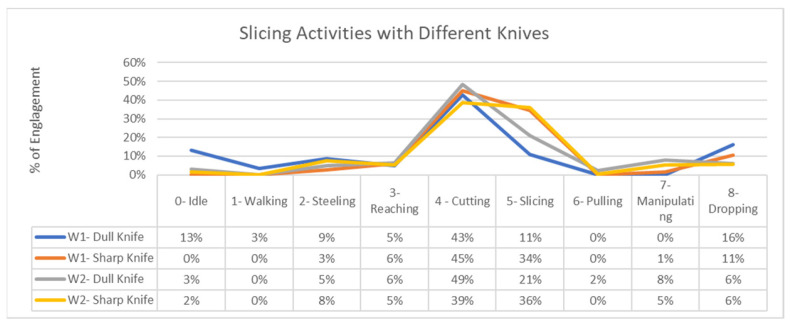
Comparison of the engagement in slicing.

The evaluation of participants’ proficiency suggests that there is a correlation between the sharpness of a knife and performance, as their body movement changed accordingly. To prove this, we conducted a preliminary analysis to demonstrate that the proposed framework can be used for recognising work activities and analysing individualised proficiency. For this, initially, we used 12 features out of 969 features (right-hand acceleration features) with dull and sharp knives for both participants. Part of the results are presented in Figure 16.

**Figure 17:**
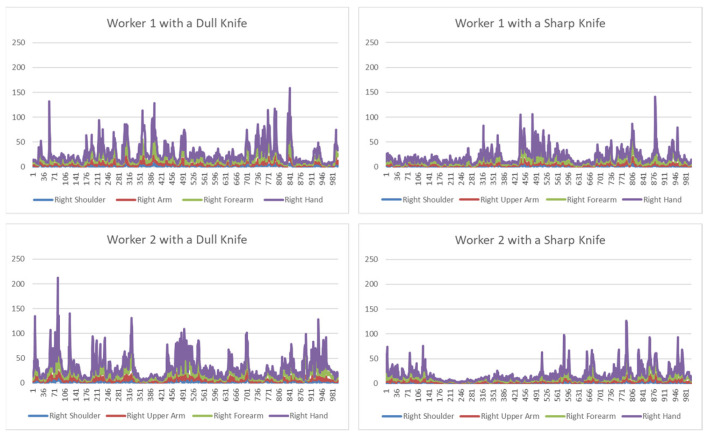
Comparison of the accelerations of the right hand.

The top two graphs in Figure 17 depict the magnitude of acceleration for the right hand of worker 1 with different knives, and the bottom two graphs show the acceleration of the right hand of worker 2 with knives. Here, data from sensors from the right shoulder, right upper arm, right forearm, and right hand are presented, which represents their prominent hand acceleration during cutting while boning the meat. From the analysis, it has been observed that for both, workers’ acceleration of their hand increased with a dull knife, which indicates that their performance decreased and their effort level increased because of the knife’s lack of sharpness.

## 6. Discussion of Skill Proficiency

Section 5 presents the performance of the proposed model of work activity classification, as well as comparisons of the body movements of the workers, to understand work proficiency. In addition, with the use of the IoT-GAN-ML-based classification model, we also identified the idle times for each worker, the wait times while sharpening the knife, and total active time contributing to work, and, most importantly, we compared the proficiency for various dimensions. Hence, similarly, the proposed framework can be used to detect the effectiveness of tools used during work, unusual body movements, and recommend areas for improvement via training for real-life work activities. The above findings prove that the proposed framework can assess proficiency in work activities, which in turn can contribute to personalising and digitalising the training of workers. Therefore, if sensor data are continuously fed into the proposed model, the proposed DigitalUpSkilling framework is capable of producing activity recognition and proficiency measurement with more features provided by the sensors, which will suit any dynamic work or sports environment with real-life physical work or sports activities.

**Figure 18:**
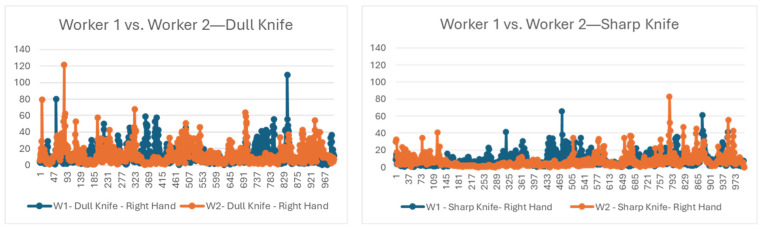
Comparison of the accelerations of the right-hand.

In addition to work proficiency, identifying body movements such as unusual acceleration or other movement during work can help us prevent potential work-related injury from repetitive limb movements [20]. Studies show that there are ergonomic risk factors for people who are engaged in repetitive tasks in physical-activity-based industries. The risk can come from excessive force, stressful posture, repetitive movement, lifting, climbing, etc. [19]. In the extended experiment, initially, we compared the accelerations of the participants’ body parts when performing a similar activity to determine the difference in acceleration for the same activity. For instance, Figure 18 shows a comparison of the accelerations of both workers’ hands. Here, we observed that, previously, both workers showed a significant increase in acceleration for different parts of their hands. However, it was found that worker 1 was consistently generating more acceleration and, thus, rapid hand movement than worker 2, regardless of the knife’s sharpness.

**Figure 19:**
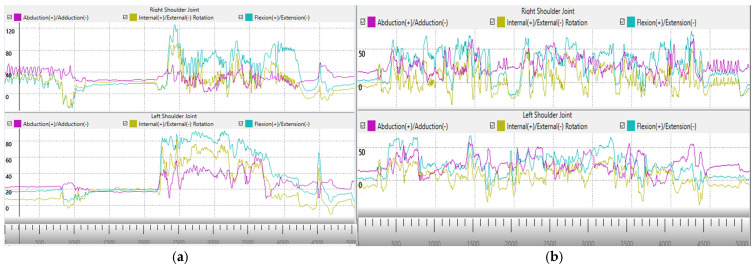
Comparisons of abduction, rotation, and flexion of the right shoulder during boning activities: (**a**) worker 1; (**b**) worker 2.

Furthermore, we compared the joint angles and abduction of both participants with similar knives for boning activity represented by digital twins. The live representations from sensor data in Figure 19 show that the right and left shoulder joint movements, such as abduction and rotation, especially flexion/extension of worker 1, exhibited greater variability and amplitude, especially during peak activity periods. Worker 2 had more controlled and less intense activity for both the right and left shoulder joints and showed smoother and more consistent movement patterns with lower variability across the same joint movement parameters. Shoulder flexion is the forward motion of the arm, usually lifting the arm in front of the body. Increased shoulder flexion variability is a sign of irregular or excessive body motions, especially while doing repetitive tasks like those in a meat processing facility.

Both results, acceleration from sensor data (Figure 18) and abduction graph from digital twins (Figure 19), demonstrate that worker 2 had smoother hand movements than worker 1. These are the attributes that are usually difficult to oversee by a human trainer. These findings will help both the trainer and trainee to focus on particular areas for improvement to further improve work proficiency, as well as to practice healthy body movements during work. Hence, the proposed framework can provide better input for trainees and trainers and improve the current approach to training.

Here the comparisons were conducted based on 8–12 features. With the digital twins and a hybrid GAN-ML model, the DigitalUpSkilling framework can explore results from many more features from a total of 969 features for a more comprehensive analysis. This data can be used to train an ML model to identify unusual body movements and categorise those that are potentially harmful. These findings can be utilised in personalised rehabilitation exercises, assuring optimal recovery and lowering the risk of injury [78,79]. Thus, these findings using the DigitalUpSkilling framework have the potential to be used in rehabilitation and sports to prevent injuries during training and exercise [80].

## 7. Conclusions and Future Directions

In this study, we proposed DigitalUpSkilling, a framework for digital and personalised worker training. The framework advances current IMU-based activity recognition by integrating digital twins with a combination of GAN advanced machine learning techniques, particularly focusing on addressing challenges in real-time body movement through digital twins and data augmentation for small datasets. Unlike traditional approaches, we utilised a hybrid GAN-ML model that enhances data through GAN-based synthetic data generation, combined with SMOTE and ENN to handle class imbalance and remove noise, ultimately improving classification accuracy. Additionally, the model leverages sensor fusion techniques and non-linear feature transformation with Leaky ReLU and Dense (128 units) layers, resulting in refined body movement assessment. The integration of these methods demonstrates improved robustness in varying work environments with limited data availability. To illustrate the framework and its benefits, the paper presented a case study on meat processing. The study’s results show that the proposed hybrid GAN-ML-based work activity recognition can recognise work activities using IoT sensor data with an accuracy of over 90%.

The study also included preliminary work skill proficiency assessments, such as acceleration of hand, rotations and movement smoothness, using 12 out of 969 possible features obtained from wearable sensors. While a trainer’s observation can consider only a few work-skill proficiency assessment parameters, the proposed models can potentially take into account other important features from these 969 features, achieving a more comprehensive and specific skill proficiency assessment. This study can be further improved to analyse proficiency in terms of body posture, and smoothness of movement during work activities from abduction data, which would not only contribute to work proficiency but also contribute to preventing injurious movements during work activities.

The future areas for improvement in this paper are as follows: first, both of the participants in the study were professional trainers in the meat industry, so there was no significant difference in skill proficiencies and body movements. In future work, we intend to include participants with more diverse skill proficiencies. Moreover, we also plan to apply the use of semi-supervised learning models and deep learning models for activity recognition and skill proficiency measurement. Finally, we aim to upgrade the DigitalUpSkilling framework to include more metrics from body movements, such as calculating jerk metrics and RULA scores from digital twins, which would be beneficial to other areas of physical training beyond specialised work tasks, such as sports, post-operative rehabilitation, and prevention of occupational injuries.

## Figures and Tables

**Table 1:** Actions and activities.

Actions and Activities	Names
Work Activities	Idleness, walking, cutting, reaching, slicing, dropping
Single Actions	body locomotion: sit, stand, walk, bend left-hand actions: spread, reach, open, close, move, unlock, hold, cut, spread, release, drop, pick, throw right-hand actions: spread, reach, open, close, move, unlock, hold, cut, spread, release, drop, pick, throw left-leg actions: still, move, spread, straighten, bend, lift right-leg actions: still, move, spread, straighten, bend, lift

**Table 2:** Activity Labelling—Boning.

Activity	Label	Observation
Idle	0	The participant keeps a hand stationary and waits for the next piece to arrive in the carcass.
Walking	1	The participant walks to get the next piece of meat in the carcass or to move around.
Steeling	2	The participant sharpens the knife with sharpening tools; to do this, they use both hands.
Reaching	3	The participant reaches for a new piece of meat from the carcass.
Cutting	4	Using the knife, the participant turns a large piece of meat into smaller pieces.
Dropping	5	The participant grabs a small piece of separated meat and throws it away on the conveyor belt.

**Table 3:** Activity Labelling—Slicing.

Activity	Label	Actions Description
Idle	0	The participant keeps a hand stationary and waits for the next piece to arrive in the carcass.
Walking	1	The participant walks to get the next piece of meat in the carcass or to move around.
Steeling	2	The participant sharpens the knife with sharpening tools; to do this, they use both hands.
Reaching	3	The participant reaches for a new piece of meat on the belt or table.
Cutting	4	The participant turns a large piece of meat into a smaller piece with the help of a knife.
Slicing	5	The participant cuts fats from a meat piece.
Pulling	6	The participant rips away fat/meat from the meat piece.
Placing/Manipulating	7	The participant manipulates the meat placement or pinches the meat.
Dropping	8	The participant grabs a small piece of separated meat and throws it away.

**Table 4:** Accuracy comparison with sensor data from different body parts.

Sensors	Activity	Velocity	Magnitude	Pitch and Roll	Velocity + Magnitude + Pitch and Roll
Right Hand (4 Sensors)	Boning	0.7371	0.8372	0.9936	0.9921
	Slicing	0.4972	0.5648	0.9779	0.9737
Both Hand (8 Sensors)	Boning	0.8062	0.9158	0.9984	0.9992
	Slicing	0.5275	0.7359	0.9923	0.9897
Full Body (17 Sensors)	Boning	0.8372	0.8713	0.9984	0.9984
	Slicing	0.5563	0.5988	0.9962	0.9962

**Table 5:** Accuracy comparison with data fusion.

Data	Activity	Accuracy	Precision	Recall	F-Score
3D acceleration	Boning	0.8362 ± 0.0011	0.8516 ± 0.0048	0.8362 ± 0.0011	0.8032 ± 0.0101
	Slicing	0.596 ± 0.0397	0.69 ± 0.0346	0.596 ± 0.0397	0.5188 ± 0.0604
Magnitude	Boning	0.8682 ± 0.0031	0.8651 ± 0.0056	0.8682 ± 0.0031	0.8531 ± 0.0087
	Slicing	0.6213 ± 0.0225	0.6854 ± 0.0082	0.6213 ± 0.0225	0.566 ± 0.0356
Pitch and roll	Boning	0.9979 ± 0.0005	0.998 ± 0.0004	0.9979 ± 0.0005	0.9979 ± 0.0005
	Slicing	0.9961 ± 0.0001	0.9962 ± 0.0001	0.9961 ± 0.0001	0.9961 ± 0.0001
3D Acceleration, magnitude, pitch and roll	Boning	0.9982 ± 0.0002	0.9982 ± 0.0002	0.9982 ± 0.0002	0.9982 ± 0.0002
	Slicing	0.9963 ± 0.0001	0.9964 ± 0.0000	0.9963 ± 0.0001	0.9963 ± 0.0001
3D Acceleration, magnitude, pitch and roll, centre of mass	Boning	0.9978 ± 0.0006	0.9978 ± 0.0006	0.9978 ± 0.0006	0.9978 ± 0.0006
	Slicing	0.9962 ± 0.0009	0.9962 ± 0.0009	0.9962 ± 0.0009	0.9962 ± 0.0009

## Data Availability

Data and results are included in the article. Further inquiries can be directed to the corresponding author/s.
